# Genotype GI.6 Norovirus, United States, 2010–2012

**DOI:** 10.3201/eid1908.130445

**Published:** 2013-08

**Authors:** Eyal Leshem, Leslie Barclay, Mary Wikswo, Everardo Vega, Nicole Gregoricus, Umesh D. Parashar, Jan Vinjé, Aron J. Hall

**Affiliations:** Centers for Disease Control and Prevention, Atlanta, Georgia, USA

**Keywords:** Norovirus, GI.6 genotype, outbreak, surveillance, viruses, United States, gastroenteritis

## Abstract

We report an increase in the proportion of genotype GI.6 norovirus outbreaks in the United States from 1.4% in 2010 to 7.7% in 2012 (p<0.001). Compared with non-GI.6 outbreaks, GI.6 outbreaks were characterized by summer seasonality, foodborne transmission, and non–health care settings.

Noroviruses are the leading cause of epidemic gastroenteritis, including foodborne outbreaks, and a major cause of sporadic gastroenteritis in the United States (*1*–*3*). Hospitalizations and deaths associated with norovirus infection occur most frequently among elderly persons, young children, and immunocompromised persons (*2*,*4*). Noroviruses can be divided into at least 5 genogroups (GI–GV) and at least 35 genotypes. Human disease is primarily caused by GI and GII noroviruses, and most norovirus outbreaks are caused by genotype GII.4 viruses (*5*). During the past decade, new GII.4 strains have emerged every 2–3 years, replacing previously predominant GII.4 strains (*6*–*8*). GI noroviruses are relatively uncommon, and systematic descriptions of GI outbreak epidemiology and characteristics are scarce (*9*). Before 2010, genotype GI.6 noroviruses were rarely reported in the United States; <5 GI.6 outbreaks were reported each year to the Centers for Disease Control and Prevention (J. Vinjé, pers. comm.). We report the emergence of GI.6 norovirus as a cause of outbreaks in the United States and discuss its effect on public health.

## The Study

Since 2009, the Centers for Disease Control and Prevention has operated 2 surveillance systems for norovirus outbreaks in the United States: CaliciNet and the National Outbreak Reporting System (NORS). CaliciNet is an electronic laboratory surveillance network that collects information on genetic sequences of noroviruses implicated in outbreaks (*5*). As of 2011, public health laboratories in all 50 states are either certified members of CaliciNet or submit norovirus-positive specimens to 1 of 5 regional CaliciNet Outbreak Support Centers. NORS is an electronic surveillance system for reporting all enteric disease outbreaks, regardless of etiology or mode of transmission (*3*). Data reported in NORS include outbreak characteristics, demographics, symptoms, implicated exposures, clinical outcomes, and etiologies

**Figure 1:**
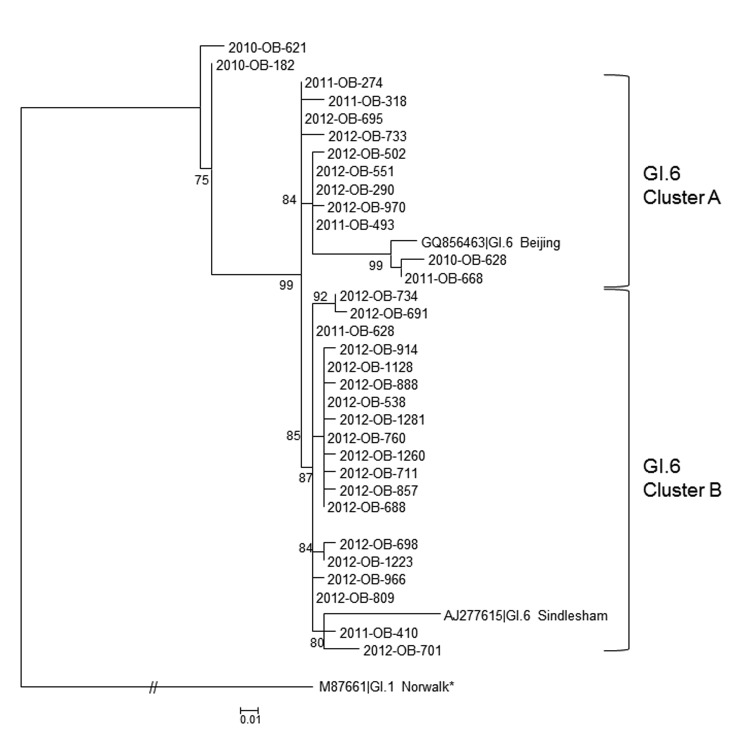
Phylogenetic typing results for GI.6 noroviruses, United States, 2010–2012. Representative outbreak nucleotide sequences were genotyped by region D (*5*). Sequences were downloaded, trimmed, and analyzed as described (*5*). In brief, a 3-parameter model, TPM1, with equal frequencies and invariable sites was run in PhyML 3.0 (www.atgc-montpellier.fr/phyml/binaries.php) as determined by jModel test by using the corrected Akaike information criterion. The best of 5 random trees was used to start the analysis, and the approximate likelihood ratio test was used for branch support. GI.6 reference sequences (GenBank accession nos. GQ856463| GI.6 Beijing and AJ277615| GI.6 Sindlesham) were included. Two clusters of genetically related outbreaks (cluster A and cluster B) are marked by brackets. *The distance of GI.1 Norwalk to the nearest GI.6 cluster is 2.29 substitutions per site. Scale bar indicates nucleotide substitutions per site for the phylogenetic tree.

We identified GI.6 outbreaks with a first illness onset date during January 1, 2010–December 31, 2012, from CaliciNet and linked them to NORS by using unique outbreak identification numbers. Supplemental information derived from NORS included mode of transmission, outbreak setting, and patients’ demographic features and clinical outcomes. State health departments were queried about outbreaks that could not be linked to NORS and requested to provide such supplemental information directly. Annual variation in GI.6 outbreaks was assessed by χ^2^ test for trend, and GI.6 seasonality was identified on the basis of visual examination of trends over time and compared with non-GI.6 seasonality by using Mid-P exact test. Norovirus-positive specimens were typed by using region D sequence analysis (*5*) (Figure 1).

**Figure 2:**
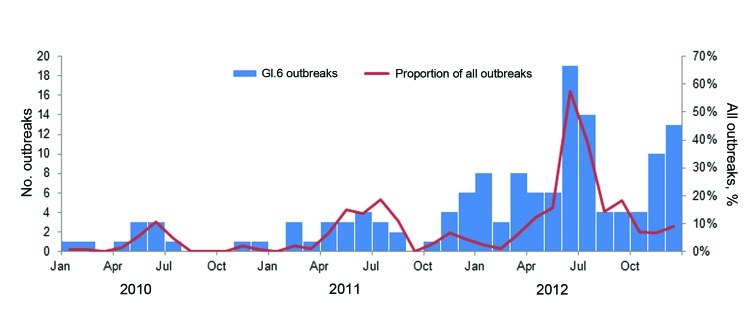
GI.6 norovirus outbreaks reported through CaliciNet, United States, 2010–2012.

A total of 141 GI.6 outbreaks in 27 states were identified over the 3-year study period. During 2010 and 2011, causitive strains for 12 (1.4%) of 879 and 30 (3.9%) of 760 outbreaks, respectively, reported through CaliciNet were typed as GI.6. During 2012, 99 (7.7%) of the 1,279 norovirus outbreaks reported through CaliciNet tested positive for GI.6, indicating a significant increase in genotype GI.6 outbreaks over the 3-year period (Figure 2; p<0.001). During 2010–2012, a total of 66 (46.8%) of 141 GI.6 outbreaks occurred during April–July, compared with 382 (13.8%) 2,777 non-GI.6 outbreaks (p<0.001).

The most commonly identified mode of transmission was person-to-person, which occurred in 81 (57.4%) GI.6 outbreaks (Table 1). Foodborne transmission was more frequent among GI.6 than among non-GI.6 outbreaks reported to CaliciNet during the same period (rate ratio [RR] 1.77, 95% CI 1.25–2.51). Waterborne transmission also was more common in GI.6 outbreaks; the 2 waterborne GI.6 outbreaks occurred during June and July.

**Table 1:** Mode of transmission and setting for norovirus outbreaks reported through CaliciNet, United States, 2010–2012

Characteristic	GI.6 outbreaks, no. (%) n = 141	Non-GI.6 outbreaks, no. (%), n = 2,777	Rate ratio (95% CI)
Mode of transmission			
Person-to-person	81 (57.4)	1,701 (61.2)	0.94 (0.81–1.08)
Foodborne	28 (19.9)	311 (11.2)	1.77 (1.25–2.51)
Waterborne	2 (1.4)	2 (0.1)	19.70 (2.80–138.80)
Other	0	15 (0.5)	Not applicable
Unknown	30 (21.3)	748 (26.9)	0.79 (0.57–1.09)
Setting			
Long-term–care facilities	51 (36.2)	1,715 (61.8)	0.59 (0.47–0.73)
Schools or daycare centers	23 (16.3)	198 (7.1)	2.29 (1.54–3.40)
Restaurants	20 (14.2)	258 (9.3)	1.53 (1.00–2.33)
Parties and events	17 (12.1)	153 (5.5)	2.19 (1.37–3.51)
Cruise ships	4 (2.8)	71 (2.6)	1.11 (0.41–3.00)
Hospitals	1 (0.7)	109 (3.9)	0.18 (0.03–1.29)
Other non–health care settings	20 (14.2)	202 (7.3)	1.95 (1.27–2.99)
Unknown	5 (3.5)	71 (2.6)	1.39 (0.57–3.38)

The most commonly reported outbreak setting was long-term–care facilities, representing 51 (36.2%) outbreaks. GI.6 outbreaks were reported less frequently in health care–related settings (hospitals and long-term–care facilities) than were non-GI.6 outbreaks (36.9% vs. 65.7%; RR 0.56, 95% CI 0.45–0.70).

GI.6 outbreaks accounted for 4,375 reported illnesses, with a median of 22 (range 2–178) reported illnesses per outbreak. Supplementary demographic and clinical outcome information was available for 66 (46.8%) outbreaks, comprising 2,220 reported illnesses. Data on hospitalization and death were provided for most (>79.0%) of these illnesses; other patient characteristics were reported less frequently (Table 2). Most (52.2%) patients were male, and 22.2% were >75 years of age; 1.4% of GI.6 outbreak patients were hospitalized, and 0.2% died.

**Table 2:** Characteristics of case patients in outbreaks of acute gastroenteritis caused by GI.6 norovirus, United States, 2010–2012*

Characteristic	No. affected/total (%)
Sex	
M	465/890 (52.2)
F	425/890 (47.8)
Age, y	
<5	8/802 (1.0)
5–9	30/802 (3.7)
10–19	345/802 (43.0)
20–49	166/802 (20.7)
50–74	75/802 (9.4)
>75	178/802 (22.2)
Outcome	
Outpatient visit	50/946 (5.3)
Emergency department visit	14/966 (1.4)
Hospitalization	24/1,753 (1.4)
Death	3/1,762 (0.2)

*Includes 66 GI.6 outbreaks (2,220 ill persons) for which at least partial supplementary data were available through the National Outbreak Reporting System or directly from state health departments.

Molecular typing data demonstrated that GI.6 viruses can be grouped into 2 clusters (Figure 1), with earlier outbreaks occurring deeper in the tree. One of the earliest occurring outbreaks in cluster A (2011-OB-274) occurred in Tennessee in February 2011 and involved a conference with 8,000 attendees and 143 reportedcases in persons from 12 states.

## Conclusions

We detected an increase in GI.6 outbreaks in the United States since 2010, with peak activity during summer 2012. Summer seasonality, foodborne transmission, and non–health care settings characterized GI.6 outbreaks, compared with non-GI.6 outbreaks reported through CaliciNet. Noroviruses are the most common cause of gastroenteritis outbreaks, and although GI.6 noroviruses remain responsible for a relatively small proportion of all reported norovirus outbreaks, they have unique characteristics and public health implications that differ from those of more common genotypes.

During 2010–2012, genotype GII.4 consistently represented most (70%) of the norovirus outbreaks reported through CaliciNet (J. Vinjé, pers. comm.). Therefore, the unique characteristics of GI.6 outbreaks described here primarily reflect differences between GI.6 and GII.4 noroviruses.

The absolute number of outbreaks and the proportion of outbreaks caused by GI.6 noroviruses peaked during April–July. This summer seasonal pattern contrasts with the overall winter seasonality of norovirus outbreaks driven primarily by winter surges in GII.4 norovirus activity (*3*,*6*–*8*). A study of GI norovirus outbreaks in Australia demonstrated peak outbreak activity during their summer months, compared with a late winter peak for GII norovirus outbreaks (*9*). In a previous study in the United States, the highest number of GI outbreaks occurred during April–May, but no apparent seasonality was noted (*7*).

Comparisons of hospitalization and death rates reported during GI.6 outbreaks with those observed in recent outbreaks caused by the emergent GII.4 Sydney strain (*8*) indicated slightly lower rates of hospitalization (1.4% vs. 2.2%; RR 0.63, 95% CI 0.39–1.02) and death (0.2% vs. 0.4%; RR 0.44, 95% CI 0.12–1.62). This observation may reflect a previously described association of GII.4 outbreaks with severe outcomes (*10*).

Region D typing data presented in this study indicates 2 clusters of GI.6 noroviruses in the United States. In February 2011, an outbreak among persons from multiple states occurred at a conference in Tennessee; this outbreak might have been a dissemination event for GI.6 activity because outbreaks of genetically related GI.6 noroviruses belonging to the same cluster occurred later in several of the states in which the conference attendees resided. However, more sequence information from the complete open reading frame (ORF2) or the hypervariable region of the protruding domain (P2) is needed to confirm possible links among the outbreaks (*5*).

Our study has several limitations. These include incomplete linkage of outbreaks reported in CaliciNet to outbreak reports in NORS and the resulting gaps in data on transmission mode and setting, as well as low rates of reporting of demographic characteristics, symptoms, and clinical outcomes. These limitations preclude direct comparison of GI.6 outbreak characteristics with characteristics of outbreaks linked to other genotypes. Efforts to improve reporting rates and integration between CaliciNet and NORS are under way (*8*).

Noroviruses are a diverse group of pathogens with varied characteristics. Continued surveillance for norovirus outbreaks through CaliciNet and NORS will enable further assessment of the public health implications and significance of emergence of relatively rare noroviruses, such as GI.6. Proper hand hygiene, environmental disinfection, and isolation of ill persons remain the mainstays of norovirus prevention and control (*11*).
